# Upsetting the Dogma: Germline Selection in Human Males

**DOI:** 10.1371/journal.pgen.1002535

**Published:** 2012-02-16

**Authors:** James F. Crow

**Affiliations:** Laboratory of Genetics, University of Wisconsin, Madison, Wisconsin, United States of America; University of Wisconsin–Madison, United States of America

As early as 1912, Wilhelm Weinberg, the visionary human geneticist, noted that infants with achondroplasia (short-limbed dwarfism) tended to be born late in their sibship [1]. From this he made the astonishing intellectual leap to the conclusion that this might signal a mutational origin for these infants' condition. This was an amazing insight considering the limited knowledge of mutation at that time. By the year 2000, the big picture seemed clear [2]: it was known that there are many more pre-meiotic cell divisions in the ancestry of a sperm than of an egg, and this seemed like a sufficient explanation for the much higher male than female mutation rate.

Yet there were exceptions. Some chromosome changes, including small duplications and deletions, seemed to have different rules of inheritance. And there were a few conditions, notably those associated with the genes *FGFR2*, *FGFR3*, and *RET*, that were more extreme: the new mutations were almost exclusively in males. Furthermore, there was a large increase in mutations with paternal age. It appeared as if these three loci, and very likely others, were marching to a different drummer [2].

The first major breakthrough came from the work of Andrew Wilkie, Anne Goriely, and their colleagues [3]. They studied *FGFR2*, which mutates to cause Apert syndrome. Using an enzyme that cuts the normal but not the mutant DNA at the relevant site, they identified *FGFR2* mutations in sperm from normal males. The overall mutation rate, as inferred from sperm studies, agreed with the incidence data for Apert syndrome. But the distribution of mutants was quite different, somewhat resembling a Delbruck–Luria jackpot. Delbruck and Luria studied mutations that occur in a growing culture of bacterial cells: if a mutation occurs in a multiplying colony, the mutation is multiplied, leading to a cluster of mutations, or jackpot (the size of the jackpot depends on the number of cell divisions since the mutation occurred).

Definitive proof came with a study of the location of the mutants on one or the other of the two members of a chromosome pair, identified by marker genes. Rather than a binomial distribution, these showed a large excess of identical alleles. The authors inferred that there must be some sort of pre-meiotic selection favoring mutations. This was a remarkable result, considering the rarity of such a process in various species and the prevailing dogma that no such thing occurs in mammals. Such selection immediately supplied an explanation of the high “mutation rate” and the paternal age effect.

**Figure 1:**
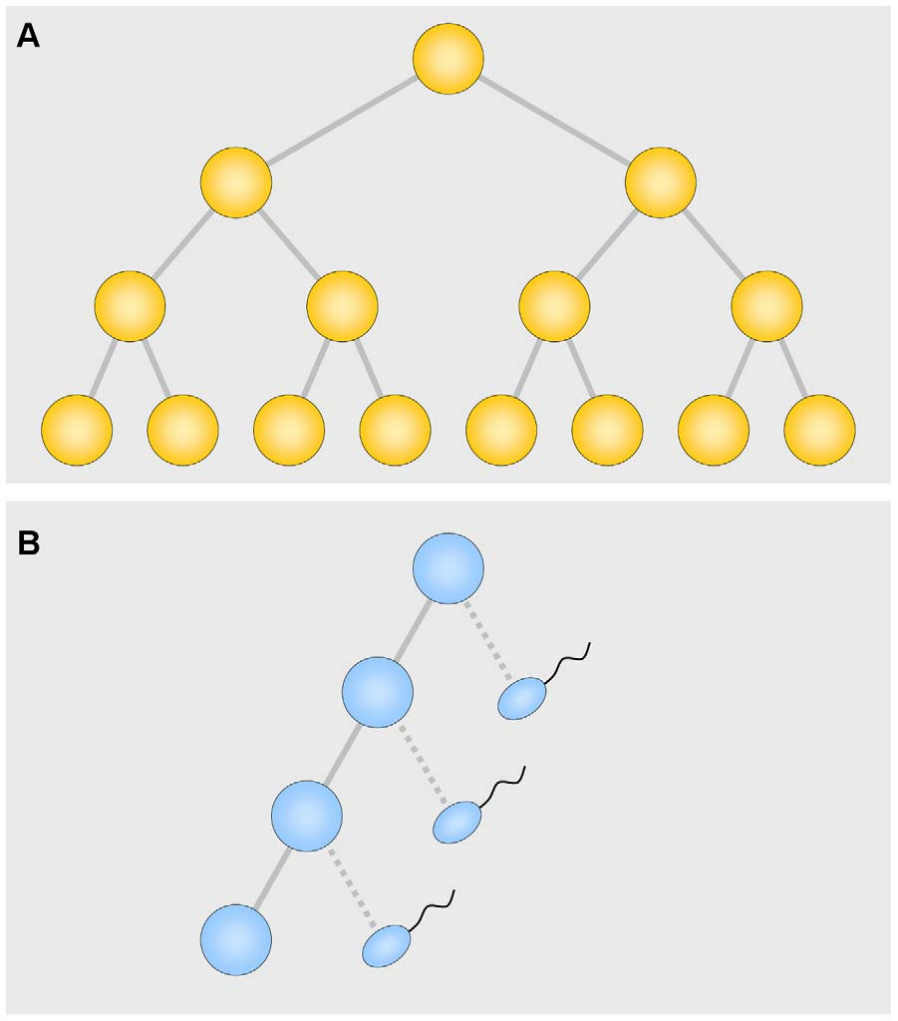
Symmetrical (A) and asymmetrical (B) divisions. In the asymmetrical divisions each cell produces one daughter like itself and one that, after 6 divisions, develops into a sperm cell. Since there are many more asymmetrical divisions, especially in older men, most of the mutations occur during the period of asymmetrical divisions.

An attractive idea for the nature of the selection is that among the asymmetrical spermatogonial divisions, producing one daughter cell like the parent and one that develops into a sperm cell, occasional symmetrical divisions (two daughter cells like the parent) occur (Figure 1). These, of course, confer a large selective advantage by producing twice as many cell descendants. Arnheim and his colleagues attacked the problem head-on, studying the mutation underlying Apert syndrome [4] and, in this issue of *PLoS Genetics*, the mutation involved in multiple endocrine neoplasia type 2B (MEN2B) [5]. In the current study, they divided several normal testes into 192 segments each. The striking result was that an individual segment usually had no or only a few mutations among normal sperm, but that an occasional segment had a very large number. Thus the mutations occur in clusters, precisely as a selection hypothesis would predict. The number of clusters increases with age. By fitting adjustable parameters to the data, Arnheim and his associates found that a one percent probability of a symmetrical division best fits the data. This adds very strong support to the idea that “selection” is nothing more than symmetrical division producing two daughter cells instead of one. This explains not only the high mutation rate, but the strong paternal age effect. Other less appealing mechanisms are not ruled out, however. At first the result for MEN2B seemed erratic for the very old men, but a correction for age-related cell death was sufficient to remove the discrepancy.

This beautiful result immediately leads to several questions. How many more loci are there that use this device? There are a number of examples in biology of easy transition between symmetrical and asymmetrical division. Why are examples, especially in higher vertebrates, so rare? The symmetrical type may cause a harmful effect. If the zygotic property of a gametically favored trait is harmful, the harmful effect may well prevail. As Haldane once said: “Clearly a higher plant species is at the mercy of its pollen grains” [6]. Even a small difference in pollen tube growth, or in our example, a small difference in number of symmetrical divisions, may cause the trait to prevail, to the detriment of the species. If the process were frequent it could be devastating. So nature must have invented mechanisms to reduce the frequency of such a change.

The MEN2B system offers a promising way to approach this and other equally interesting problems because so much is known in the mouse: for example *RET*, the gene mutated in MEN2B, is necessary for spermatogonial self-renewal. The materials and technique are ready. We can look forward to much deeper biochemical and cytological knowledge of spermatogenesis and the ways in which it can be modified.

Asymmetrical division is one of nature's cleverest devices. In a human male it yields a constant daily supply of sperm. Yet mutation to symmetry occurs in a number of biological systems. The process of asymmetrical division is in constant danger of sabotage by mutants waiting to beat it.
